# Identifying the Need to Discuss Infertility Concerns Affecting Testicular Cancer Patients: An Evaluation (INDICATE Study)

**DOI:** 10.3390/cancers13030553

**Published:** 2021-02-01

**Authors:** Esmée M. Krouwel, Thijs G. Jansen, Melianthe P. J. Nicolai, Sandra W. M. Dieben, Saskia A. C. Luelmo, Hein Putter, Rob C. M. Pelger, Henk W. Elzevier

**Affiliations:** 1 Department of Urology, Leiden University Medical Center, 2333 ZA Leiden, The Netherlands; jansen.tg92@gmail.com (T.G.J.); r.c.m.pelger@lumc.nl (R.C.M.P.); h.w.elzevier@lumc.nl (H.W.E.); 2 Department of Medical Decision Making, Leiden University Medical Center, 2333 ZA Leiden, The Netherlands; 3 Department of Urology, Netherlands Cancer Institute-Antoni van Leeuwenhoek Hospital, 1066 CX Amsterdam, The Netherlands; m.nicolai@nki.nl; 4 Department of Gynaecology, Leiden University Medical Center, 2333 ZA Leiden, The Netherlands; s.w.m.dieben@lumc.nl; 5 Department of Medical Oncology, Leiden University Medical Center, 2333 ZA Leiden, The Netherlands; s.a.c.luelmo@lumc.nl; 6 Department of Medical Statistics, Leiden University Medical Center, 2333 ZA Leiden, The Netherlands; h.putter@lumc.nl

**Keywords:** fertility preservation, testicular neoplasms, quality of life, practice patterns

## Abstract

**Simple Summary:**

Testicular cancer is the most common malignancy in young males affecting the ability to father children. It’s important that effects on fertility are discussed before starting treatment so patients are aware of the risks and their options. The objective of our study was to evaluate the manner in which men with testicular cancer are counselled about implications on fertility and the possibility of semen preservation. Furthermore, we aimed to evaluate satisfaction with provided information and to identify reproductive concerns. In a sample of 201 patients, one out of ten patients reported not to be informed about the risk of subfertility. Sperm banking was performed by 41.3%, of which 13 men made use of preserved sperm, resulting in paternity for 7 men. The subjects fertility and semen preservation need to be broached promptly after diagnosis of testicular cancer because they cause dissatisfaction with care and grief if fertility problems occur afterwards.

**Abstract:**

Men with testicular cancer (TC) risk impaired fertility. Fertility is a major concern for TC patients due to diagnosis in almost always reproductive ages and high overall survival. This study assessed counselling in regards to the risk of impaired fertility and sperm cryopreservation. A cross-sectional survey was performed on 566 TC patients diagnosed between 1995–2015. Of the 566 survivors, 201 questionnaires were completed (35.5%). Eighty-eight percent was informed about possible impaired fertility, 9.5% was not informed. The majority (47.3%) preferred the urologist to provide information. Collecting sperm was troublesome but successful for 25.6%, 4.8% did not succeed in collecting sperm. The reasons were high pressure due to disease, pain after surgery and uncomfortable setting. Due to impaired fertility, 19% of the respondents reported grief and 9.3% stated as being less satisfied in life. Sperm cryopreservation was performed by 41.3% (*n* = 83). One third (*n* = 63, 31.3%) had children after treatment, of which 11.1% made use of preserved sperm (*n* = 7). The results of this survey indicate the importance of timely discussion of fertility issues with TC patients. While being discussed with most men, dissatisfaction and grief may occur as a result of impaired fertility and a lack of counselling. Overall, 6.5% made use of cryopreserved sperm (*n* = 13). Men prefer their urologist providing counselling on fertility.

## 1. Introduction

Testicular cancer (TC) is the most common type of cancer affecting men between 15 and 44, particularly in white Caucasian populations [1]. Over the past decades, in industrialised countries, and especially in Northern and Western Europe, TC incidence has increased and continues to rise [2]. In the Netherlands, the incidence has doubled in the past two decades, with over 800 men diagnosed every year [3]. At diagnosis, patients with TC are staged according to the presence and site of metastatic lesions and the serum levels of tumour markers. Most patients diagnosed with TC are primarily treated with orchiectomy, and subsequent therapy depends on the tumour histology, stage and prognosis group [4]. Adjuvant treatment may involve surveillance, chemotherapy, nerve-sparing retroperitoneal lymph-node dissection (RPLND) or radiotherapy.

Cure rates for non-metastatic TC are excellent and even for metastatic TC patients are the chances of cure and long-term survival high because of greatly effective chemo- and radiotherapy [5]. High five-year-survival rates make quality of life important to consider in the treatment of TC, as many TC patients survive for decades after being diagnosed [5,6]. TC, together with poor semen quality, hypospadias, and undescended testis, is part of the testicular dysgenesis syndrome [7]. Gonadal dysfunction with subnormal testosterone levels in TC survivors is common after treatment, which has a major impact on quality of life [8,9]. Moreover, treatment of TC can either temporarily or permanently impair fertility [10]. Chemotherapy and radiotherapy are likely to impair spermatogenesis and RPLND may impact ejaculatory function [11]. Compared to the normal population, fertility decreased by 30% in TC patients after treatments, radiotherapy has the most deleterious effects [10]. However, sperm abnormalities and Leydig cell dysfunction are often already present in TC patients prior to orchiectomy due to testicular dysgenesis syndrome: 24% has azoospermia and almost 50% has oligozoospermia before surgery [12,13,14]. After orchiectomy, concentration and total sperm counts deteriorate further, especially in non-seminoma patients [13].

Taking into account pre-existing sperm abnormalities in TC patients and the chances of deteriorating fertility after treatment, fertility is a critical subject for health care providers to discuss with patients prior to commencing treatment [15,16]. Besides discussing the possibility of impaired fertility, TC patients should be offered cryopreservation prior to the start of treatment and sperm cryopreservation should be encouraged to maintain the ability to conceive a child in later life [4]. In the Netherlands, it is common practice that, within 48–72 h after diagnose, orchiectomy should follow. Health care providers are advised to discuss the risk of impaired fertility and propose cryopreservation as soon as possible after diagnose [17]. Within a short period of time after diagnosis, TC patients are confronted with not only the impact of having cancer, but also uncertainty of the possibility to have children. Sperm cryopreservation is a generally accepted method to preserve fertility in men [18]. Sperm used for cryopreservation is obtained by ejaculation or via alternative approaches in case of impairment in sperm retrieval, like percutaneous epididymal sperm aspiration (PESA) and testicular sperm extraction (TESE). Additionally, sperm cryopreservation has proven to be the most cost-effective strategy for fertility preservation in men with TC prior to undergoing chemotherapy or radiotherapy [19]. In the Netherlands, sufficient sperm banks exist to offer cryopreservation within 48 h after diagnosis, prior to orchiectomy [20]. In one out of six patients, sperm cryopreservation could be unsuccessful due to severe spermatogenesis impairment [20]. This is an important argument for performing sperm cryopreservation prior to radical orchiectomy. In these patients, testicular sperm extraction (TESE) can be performed during radical orchiectomy [13,20].

According to literature, 17% of TC patients were not offered cryopreservation [21] and barriers exist for health care providers to discuss the fertility topic [22,23]. Furthermore, a lack of information provision regarding sperm cryopreservation is identified as the biggest barrier for young male cancer patients for actually performing sperm preservation [24]. Little is known about the long-term fertility and paternity rates, and the use of preserved semen and spontaneous versus assisted paternity rates of TC survivors.

In order to evaluate fertility related issues according to men who have faced TC, a survey has been performed among TC survivors in the Netherlands. The survey included questions regarding patients’ experiences of the discussion of fertility concerns and sperm preservation, the procedure of sperm cryopreservation, the number of children and the use of preserved samples, satisfaction levels regarding information provision and reproductive concerns.

## 2. Results

From 582 invited participants, 262 responses were received (response rate 45%), of which 45 patients refused to participate. The reasons were: ‘no time’ (6), ‘no interest’ (18), ‘the diagnosis was too long ago’ (3), ‘treatment took place in another hospital’ (3), ‘bilateral orchiectomy so fertility was not an issue at the time’ (1), ‘due to my age not applicable’ (4), ‘too many requests for participation in research’ (2), ‘prefer digital questionnaire’ (1), ‘did not receive treatment’ (1) and some reported no reason (6). Excluded were patients ‘not understanding the questionnaire in Dutch’ (6), ‘mentally not capable’ (2), ’questionnaire not relevant as patient was already sterilized prior to diagnosis’ (2). Six respondents were excluded due to their age (>70 years old at time of diagnosis). These exclusions resulted in 566 eligible candidates.

A total of 201 questionnaires among the 566 eligible candidates (35.5%) have been returned. The responders and non-responders did not differ in mean age at the time of the questionnaire (44.2 years vs. 43 years) and mean age at diagnosis (33.7 years vs. 34 years). A difference was found in the mean follow-up time. The follow-up was 10.6 years for responders and 9.2 years for non-responders (*p* = 0.004, ind. sample T test).

Demographic characteristics are shown in Table 1. The mean time since diagnosis was 11 years and the mean age at diagnosis was 34 years. A majority of 81.1% was married or living together at the time of the survey and 88.6% was born in the Netherlands.

### 2.1. Information Provision Regarding Fertility Preservation

The majority of the respondents (87.6%, *n* = 176) stated to be notified about the possibility of fertility problems as a result of their treatment. Nineteen patients (9.5%, *n* = 19) stated that, as far as they remember, they have not been informed about the possibility of diminished fertility, six respondents could not remember (3%). Patients who had not been informed about possible fertility issues were mostly stage I (*n* = 15), stage II (*n* = 1) and from three patients the stage was unknown. The possibility of sperm cryopreservation was mentioned according to 77.1% of the respondents (*n* = 155); it was not mentioned according to 29 respondents (14.4%).

More than half of respondents were informed about the possibility of fertility problems by their urologist (57.7%, *n* = 116), of which 74.1% of the time in advance of the orchiectomy and 12.9% in advance of chemotherapy. Information provision regarding fertility threat by other health care providers and timing of information provision is displayed in Table 2.

Conversations regarding fertility preservation were initiated by the patient itself (*n* = 10, 9.5%), a doctor (*n* = 144, 71.6%), a nurse (*n* = 10, 5%), their partner (*n* = 2, 1%), or it had not been discussed (*n* = 4, 2%). A minority stated ‘it was not at risk according to my doctor’ (*n* = 2, 1%), one respondent said ‘I only got a referral to a fertility specialist but no explanation’ (0.5%) and one participant could not remember (0.5%). A quarter of all respondents received written information materials (*n* = 48, 23.9%) regarding fertility issues, 62.7% did not receive written information (*n* = 126). The majority prescribed the provided information as extensive (*n* = 33, 68.8%), 22.9% would have liked more extensive information (*n* = 11), two patients stated information was incomplete (4.2%). Patients found additional information on the internet (*n* = 17), through the Dutch Testicular Cancer Society (*n* = 15), the ‘KWF’ foundation (*n* = 3), Google (*n* = 10), and family and friends (*n* = 4).

### 2.2. Patient Preferred Information Provision

Participants were asked to state their preference regarding the most suitable health care provider for information provision on fertility preservation. Preferences are displayed in Table 3.

### 2.3. Treatment Related Advice Regarding Sperm Preservation

In Table 4, we display the (by participants reported) doctors’ advices regarding sperm preservation in regards to their treatments.

### 2.4. Patient Satisfaction Levels with Information Provision

Satisfaction levels regarding information provision about fertility were, respectively, very satisfied (*n* = 52, 27.7%), satisfied (*n* = 92, 48.9%), neutral (*n* = 33, 17.6%), dissatisfied (*n* = 6, 3.2%) and very dissatisfied (*n* = 5, 2.7%). Satisfaction levels regarding information provision about the possibility to perform sperm cryopreservation were, respectively, satisfied (*n* = 111, 81.6%), neutral (*n* = 11, 8.1%), and dissatisfied (*n* = 14, 10.3%).

Men that had not been informed about fertility risks and the possibility to perform sperm cryopreservation were significantly more dissatisfied with the information provision (*p* < 0.001, linear-by-linear association). Men that had not performed sperm cryopreservation reported significantly more dissatisfaction with information provision as well (*p* = 0.023, linear-by-linear association).

After finishing all treatments, 38 men reported that they had discussed their fertility concerns with a medical professional. Concerns were discussed with general practitioners (30.4%, satisfaction 85.7%), family/friends (75.5%, satisfaction 76.9%), fellow sufferers (18.6%, satisfaction 87.5%), psychologists (17.4%, satisfaction 75%) and the urologist (52%, satisfaction 56%). Twenty-nine men stated that, in retrospect, they would have wanted counselling regarding fertility concerns (14.4%).

### 2.5. Sperm Cryopreservation Procedure

To the question: “was there a possibility to choose a location for sperm production”, 65% answered affirmative (*n* = 54). Thirty-six patients reported they had been able to produce sperm in the privacy of their home, three patients obtained sperm during clinical stay in the hospital, sixty-one patients reported an attempt in the outpatient fertility clinic. The majority (69.5%, *n* = 57) was able to obtain sperm without trouble, 25.6% succeeded in collecting with some obstacles (*n* = 21), one patient reported that he was unsuccessful in producing semen due to the experienced pressure from having cancer, two patients reported not to succeed due to pressure because of collection in the hospital and one patient reported not succeeding due to pain.

Participants were asked if the costs for samples and storage fees influenced their decision. Thirty-three men reported that they were not aware of additional costs, 39 men stated that the costs would not matter and seven men reported that the costs were significant, but because of the importance, not an issue. One single patient reported that the costs influenced the decision-making and decided not to perform sperm cryopreservation.

### 2.6. Offspring before and after Testicular Cancer

Altogether, 83 men (41.3%) performed sperm cryopreservation. Thirteen out of 83 men (15.7%) that performed sperm cryopreservation reported that they made use of their sample(s), which is 6.5% from all 201 participating respondents. Seven out of 13 men reported the successful use of their sperm samples (53.8%). Five patients reported considering the usage of their sperm sample in the future (6%), 38 patients reported as not yet being sure about using the samples in the future (45.8%).

Off all the participants, 86 men (42.8%) already father children conceived before the diagnosis TC. After TC treatment, 63 men had children (31.3%). Twenty-nine men reported that they had one child, 27 men reported that they had two children, five men reported they had three children and one man reported four children after being treated for TC.

More information regarding children after TC in regards to the received treatments is shown in Table 5.

Finally, participants were asked if their wish to become parents had changed due to the TC diagnosis and treatments. According to the majority (*n* = 166, 87.4%), nothing had changed, 12 men experienced an increased wish for children (6.3%) and 12 men described a decreased wish for children (6.3%). Stage of disease was not significantly different with regards to either increased, likewise or decreased wish for children (linear-by-linear, *p* = 0.477).

### 2.7. Reproductive Concern Scale

In Table 6, the results from TC survivors scoring the validated ‘Reproductive Concern Scale’ items.

## 3. Discussion

This study provides us with a broad perspective regarding fertility concerns and sperm cryopreservation among men who survived TC. The results indicate that the majority of the respondents have been notified about the possibility of fertility problems as a result of their treatment (87.6%). However, the possibility of sperm cryopreservation was discussed with fewer respondents (77.1%). According to the respondents, the most suitable health care provider for counselling about fertility preservation is the urologist. Advices regarding sperm preservation in relation to treatments were strikingly variable, especially for the ‘orchiectomy and surveillance’ group and the ‘orchiectomy and radiation group’. In these two groups, respectively, 46.2% and 26.3% were informed that cryopreservation of sperm was not necessary (Table 4). Furthermore, varying advices were given or no advice was given at all. This is a surprising finding, realising that already before treatment, up to a quarter of TC patients are azoospermic and almost half of them have abnormal sperm counts (oligozoospermic) [13]. As for the advice regarding preservation received by patients undergoing radiation, is remarkable, as radiotherapy seems to have the most deleterious effect on fertility [10]. Written information materials regarding fertility issues were provided in less than a quarter of the respondents. This corresponds to an American survey among oncologists, where only 13.5% reported ‘always or often’ giving their patients educational materials about fertility preservation [25]. Provision of written, digital or visual information materials could be helpful, as it is a well-known phenomenon that patients often do not remember all verbally supplied information [26]. Furthermore, provision of written information could increase patient satisfaction [27,28]. In the current survey, levels of satisfaction with care could directly be correlated to the amount of information provided regarding fertility risks. Men that did not make use of sperm cryopreservation were significantly more dissatisfied. According to two thirds of respondents, sperm collection was possible on a self-chosen location. Obtaining sperm was troublesome, but eventually possible for 25.6% of respondents; 4.8% did not succeed. Reasons for troublesome collection were high pressure due to disease, pain after surgery and uncomfortable setting in the hospital. Costs regarding sperm cryopreservation and storage fees did not influence decisions for preservation according to the majority. Different results were found in the United States, where 10% noted cost as the reason for not banking sperm [21]. Costs in the USA, however, seem to be significantly higher compared to the Netherlands, which may explain the different results. Initial sampling fees in the USA nearing $1000 (€126.47 in the Netherlands) and yearly storage costs ranging from $300 to $400 (€66.29 in the Netherlands). These fees, however, are covered by every Dutch health insurance agency.

Almost one third of respondents fathered children after TC treatment. Eleven percent made use of their preserved sperm samples (*n* = 7) to procreate, six men used their sample but did not succeed in conception. This means that thirteen out of 83 men (15.7%) who banked sperm made use of their sample, this is slightly more than the average usage rate of cryopreserved sperm among male cancer patients. A systematic review of 30 studies on sperm cryopreservation in male patients with cancer showed that 8% of 11.798 patients who preserved sperm made use of their sample [29]. Success in achieving parenthood among patients who used their sperm sample was 49% and our results showed a comparable conception rate (54%).

The results of the Reproductive Concern Scale showed a rate of 35% that did not feel able to talk openly about fertility. Furthermore, 57.7% stated not feeling in control of their reproductive future. Almost a third (29.1%) was not content with the number of children they fathered. Nineteen percent of the respondents reported a little bit, somewhat or quite a bit of grief due to impaired fertility, 9.3% stated being a little bit, somewhat and some even very much less satisfied in life due to impaired fertility. These results provide insight in the long-term consequences of diminished fertility among TC survivors, emphasizing the need for optimizing fertility counselling in this group.

In most of the TC patients in this study, the experience of testicular cancer did not influence the wish to have children (87%), a small amount (6%) felt it had increased their wish to be a father, and 6% felt it decreased their wish. In a survey among young male cancer patients conducted in the United States, slightly different numbers were mentioned, as 68% of their wishes was not influenced, 16% felt an increased and 16% a decreased wish to become a father [24].

### Limitations

Limitations of this survey include the use of a partially non-validated questionnaire. However, by involving a multidisciplinary expert panel and a patient panel checking for comprehensiveness and quality, we aimed to reduce any bias resulting from the use of this questionnaire. Furthermore, it is possible that a recall bias has occurred, due to the relatively long period between diagnosis, treatment and questionnaire. In addition, with a growing attention on oncofertility in the past decade, the survey may not be representative for present-day practice. The current study was carried out single centre. Yet, as a tertiary referral centre for post radical orchiectomy follow up and treatment respondents have been primarily counselled and operated all over the region of south-west Netherlands. Consequently, our sample is expected to be representative of the surrounding peripheral hospitals as well. At 35.5%, the response rate was reasonable for a paper survey, and may have been influenced by the time from diagnosis until the survey, survey length and sensitivity of the subject (fertility concerns) [30]. However, including a sample of 201 respondents, results have to be interpreted with caution. With a significantly longer follow up time among responders vs. non-responders, it may possibly be assumed after a longer period of time the subject of fertility is more easy to reflect on for survivors.

Despite these limitations, this study is one of the first assessing TC survivors and their experience, opinions and satisfaction regarding the discussion of fertility issues and process of sperm cryopreservation. The current, relatively large sample provides us with useful insights for current practice, including preferred health care provider for counselling, satisfaction levels and the lack of provision of written information materials. Moreover, it implicates the need for further calling attention to the timely discussion of fertility preservation in TC patients among health care providers, specifically urologists. This is supported by a recent study where a cancer and fertility program was established in a large cancer centre, including clinician education, provision of resources and consultations with a fertility clinical nurse specialist. Patient satisfaction among men was significantly improved and information material was found to be particularly helpful [28]. A prospective, longitudinal study could assist in answering remaining questions regarding specific fertility concerns arising at the time of diagnosis, preferred information resources (digital; written, verbal, etc.) and whether we will meet improved reproductive outcomes in the case of sperm cryopreservation in advance of orchiectomy. Furthermore, locations for sperm collection could be improved or be facilitated at a location according to the patient’s preference more often.

## 4. Materials and Methods

### 4.1. Study Design

A cross-sectional study has been performed among TC patients at the time of January-June 2016 (*n* = 611). All TC patients diagnosed or treated at Leiden University Medical Center between 1995 and 2015 received an invitation to participate. Leiden University Medical Center is a tertiary referral centre for post radical orchiectomy treatment. By these means, orchiectomy and fertility counselling could have been performed in several peripheral hospitals from the region, after which patients have been referred to the Leiden University Medical Center for treatment and/or follow up. Men that were deceased or moved abroad have not been approached (*n* = 29), resulting in 582 eligible patients. Patients received a letter by mail explaining the study objectives and a consent form with a post-paid return envelope. Consent forms were coded in order to link respondents to an anonymized file including patients’ treatment history. Reminders were sent to non-responders after 6 weeks. When consent was provided, patients received the questionnaire accompanied by a post-paid return envelope.

### 4.2. Inclusion Criteria

Patients who are or have been under treatment of the outpatient clinic of the Urology and/or Oncology department of the LUMC with pathologically confirmed TC in their medical history. Inclusion criteria: ability to understand and fill in the questionnaire in Dutch, willingness and informed consent to participate. We excluded TC patients under 18 years old at the time of diagnosis, and deceased or patients who moved abroad. Furthermore, we excluded patients sterilized previous to diagnosis. Upper age criterium was set after checking all Dutch fertility clinics and guidelines. We found that some clinics have a maximum age of 60 years; others do not have a maximum. As we had one respondent of 79 years old explicitly stating fertility questions were not applicable, we decided to exclude respondents that were 70 years old or older.

### 4.3. Materials; Questionnaire

The questionnaire was designed by the researchers, based on the study aims and a review of the literature in the area. The Dutch validated Reproductive Concern Scale has been implemented, minimally adjusted to a male perspective [31,32]. A multidisciplinary expert panel, having experience developing surveys and having experience regarding fertility and oncology, checked the questionnaire for comprehensiveness and quality. A patient panel of two members of the Dutch Testicular Cancer Society piloted the questionnaire afterwards.

The questionnaire focussed on patients’ experience discussing fertility, cryopreservation and the quality of the information provided. Additionally, the advice given by health care providers, patients’ preference regarding discussing fertility and the experience of cryopreservation were taken into account. Lastly, the provision of information and satisfaction about testicular implants were assessed, and these results have been processed separately [33].

### 4.4. Data Analysis

Data of the questionnaires were transferred into digital files. Additional data were obtained from the oncology registration (anonymized), including age, type and staging of TC and treatment types. Demographic data of non-respondents have been compared to respondents. Data analysis was performed using SPSS (IBM SPSS Statistics for Windows, Version 25.0. Armonk, NY: IBM Corp., USA). Means of numerical demographic values and the answers to the questions have been analysed with frequency distribution. Bivariate associations between demographic information and the categorical data were calculated using the Pearson chi-square procedure and linear-by-linear association. Associations between numerical data and demographics of the respondents were analysed with the independent sample *t*-tests. Two-sided *p* values < 0.05 are considered statistically significant.

### 4.5. Ethics

Ethical approval was obtained at the local medical ethical committee, as it concerns a survey with sensitive questions. Approval was provided on 7 October 2015. A letter explaining the study and an informed consent form was provided before introducing the questionnaire.

## 5. Conclusions

Findings of this testicular cancer patients survey indicate the importance of timely discussion of fertility issues. While being discussed with most men, several TC survivors reported not having received fertility counselling or counselling with limited information. Furthermore, counselling was not always performed before orchiectomy, which is well known to negatively influence sperm sample quality. Dissatisfaction and grief may occur as a result of impaired fertility and a lack of support from healthcare providers. Overall, 6.5% made use of cryopreserved sperm, more than half of these patients achieved parenthood. Men prefer their urologist to provide information on fertility preservation. Satisfaction regarding the information offered about fertility issues varied and a there was a relative lack of written information materials, indicating room for improvement in information provision.

## Figures and Tables

**Table 1:** Demographic characteristics.

Demographic Characteristics	*n* (%)
Total eligible patients	566 (100)
Total participation rate	201 (35.5)
Mean age: 44.2 years (range 23–74)	201 (100)
Mean age at time of diagnosis 33.7 years (range 20–68)	201 (100)
Mean follow up time to questionnaire 10.6 years (range 2–21)	201 (100)
Histology	
Seminoma	101 (50.2)
Non-seminoma	96 (47.8)
Neuro-endocrine	1 (0.5)
Leydig cell tumour (malign)	3 (1.5)
Histology contralateral tumour	7 (3.4)
Seminoma	2 (28.6)
Non-seminoma	4 (57.2)
CIS	1 (14.3)
Tumor stadium	
Stage I	103 (51.2)
Stage II	29 (14.4)
Stage III	2 (1)
Stage IV	7 (3.5)
Unknown	60 (29.9)
Primary treatment	
Primary orchiectomy ^a^	200 (99.5)
Chemotherapy	1 (0.5)
Orchiectomy for contralateral tumour	7 (3.5)
Secondary	
Surveillance	48 (23.9)
Additional therapy	
Chemotherapy	96 (47.8)
+RPLND	21 (10.4)
+RT	3 (1.5)
+RPLND & RT	1 (0.5)
+Metastasectomy	3 (1.5)
+RT + Metastasectomy	1 (0.5)
Metastasectomy ^a^	1 (0.5)
Radiotherapy	27 (13.4)
Marital status	
Married/registered partnership	116 (57.7)
Relationship, living together	47 (23.4)
Relationship, living apart	13 (6.5)
Single	18 (9)
Divorced	4 (2)
Widow	1 (0.5)
Unknown	2 (1)
Educational level	
Secondary school	36 (17.9)
Secondary vocational education	50 (24.9)
Higher professional education/University	115 (57.2)
Country of birth	
Netherlands	178 (88.6)
Other (USA 1, Colombia 2, Germany 3, France 1, Indonesia 2, Iran 1, unknown 13)	23 (11.4)

^(a)^ a single patient did not primarily receive an orchiectomy as there was a burned out tumour; presenting with metastasis.

**Table 2:** Information provision regarding the possible reduced fertility.

Health Care Provider	Percentage of Discussing Fertility by Specific Provider *n* (%)	Timing	In Advance of Orchiectomy *n*(%)	In Advance of Chemotherapy *n*(%)	In Advance of Radiation *n* (%)	Other Moment *n* (%)
Urologist	116 (57.7)		86 (74.1)	15 (12.9)	5 (4.3)	10 (8.6)
Medical oncologist	93 (46.3)		10 (10.8)	64 (68.8)	5 (5.4)	14 (15.1)
Radiation oncologist	2 (1)		-	-	2 (100)	-
General practitioner	4 (2)		4 (100)	-	-	-
Oncology nurse	15 (7.5)		1 (6.7)	12 (80)	-	2 (13.3)
Fertility specialist	21 (10.4)		2 (9.5)	15 (71.4)	-	4 (19.1)

**Table 3:** Patient preferred a health care provider for counselling on treatment related fertility problems.

Preferred Health Care Provider	*n* (%)
Urologist	95 (47.3)
Oncologist	61 (30.3)
General practioner	7 (3.5)
(Oncology) nurse	11 (5.5)
All above mentioned	3 (1.5)
Specialty not relevant; doctor that is initially telling diagnosis	8 (4)

**Table 4:** Advice from physicians regarding sperm preservation in relation to treatments.

Treatment	Sperm Cryopreservation, Significant Risk Reduced Future Fertility *n* (%)	Sperm Cryopreservation, Low Risk but as a Precaution *n* (%)	No Preservation Necessary *n* (%)	Not Yet Necessary, to Reconsider if Additional Treatment is Required *n* (%)	Varying Advices Were Given *n* (%)	No Advice Given *n* (%)
Orchiectomy and surveillance	10 (25.6)	4 (10.3)	9 (23.1)	9 (23.1)	2 (5.1)	5 (12.8)
Orchiectomy and chemotherapy	52 (61.2)	13 (15.3)	3 (3.5)	4 (4.7)	5 (5.9)	8 (9.4)
Orchiectomy and radiation	6 (31.6)	5 (26.3)	3 (15.8)	2 (10.5)	2 (10.5)	1 (5.3)
Orchiectomy, chemotherapy and radiation	0	1 (50)	0	0	0	1 (50)
Orchiectomy, chemotherapy and RPLND	15 (83.3)	1 (5.6)	0	0	1 (5.6)	1 (5.6)
Orchiectomy, chemotherapy and metastasectomy	2 (100)	0	0	0	0	0
Orchiectomy, chemotherapy, radiation and metastasectomy	0	1 (100)	0	0	0	0
Abdominal tumour; chemotherapy + metastasectomy	1 (100)	0	0	0	0	0
Total	86 (51.5)	25 (15)	15 (9)	15 (9)	10 (6)	16 (9.6)

**Table 5:** Children after testicular cancer with reference to previous treatments.

Treatment	Children by Natural Conception *n* (%)	Children by Use of Preserved Sperm Sample *n* (%)	Children with Assisted Reproduction Due to Reduced Fertility of Partner *n* (%)	Children with Assisted Reproduction Due to Reduced Fertility of Patient *n* (%)	No children Yet, Attempting by Natural Conception at the Moment *n* (%)	No Children Yet, Attempting by Assisted Reproduction at the Moment *n* (%)	No Children Yet, It Was Not Possible *n* (%)	No Wish to Become a Parent (Yet) *n* (%)
Orchiectomy and surveillance	11 (22.9)	0 (0)	1 (2.1)	0 (0)	3 (6.3)	1 (2.1)	5 (10.4)	27 (56.3)
Orchiectomy and chemotherapy	26 (29.9)	2 (2.3)	2 (2.3)	1 (1.1)	2 (2.3)	0 (0)	3 (3.4)	51 (58.6)
Orchiectomy and radiation	7 (30.4)	1 (4.3)	0 (0)	0 (0)	1 (4.3)	0 (0)	0 (0)	14 (60.9)
Orchiectomy, chemotherapy and radiation	0 (0)	0 (0)	0 (0)	0 (0)	0 (0)	0 (0)	0 (0)	3 (100)
Orchiectomy, chemotherapy and RPLND	4 (19)	4 (19)	2 (9.5)	0 (0)	0 (0)	1 (4.8)	0 (0)	10 (47.6)
Orchiectomy, chemotherapy, RPLND and radiation	0 (0)	0 (0)	0 (0)	0 (0)	0 (0)	0 (0)	0 (0)	1 (100)
Orchiectomy, chemotherapy and metastasectomy	1 (33.3)	0 (0)	0 (0)	0 (0)	0 (0)	0 (0)	0 (0)	2 (66.7)
Orchiectomy, chemotherapy, radiation and metastasectomy	1 (100)	0 (0)	0 (0)	0 (0)	0 (0)	0 (0)	0 (0)	0 (0)
Abdominal tumour; chemotherapy + metastasectomy	0 (0)	0 (0)	0 (0)	0 (0)	0 (0)	0 (0)	0 (0)	1 (100)
Total	50 (26.6)	7 (3.7)	5 (2.7)	1 (0.5)	6 (3.2)	2 (1.1)	8 (4.3)	109 (58)

**Table 6:** Results of the Reproductive Concern Scale adjusted for males.

Item on the Reproductive Concerns Scale	Not at All *n* (%)	A little Bit *n* (%)	Somewhat *n* (%)	Quite a Bit *n* (%)	Very Much *n* (%)
I have concerns about my ability to have children	150 (79.4)	25 (13.2)	8 (4.2)	4 (2.1)	2 (1.1)
I am content with the number of children that I have	53 (29.1)	10 (5.5)	8 (4.4)	14 (7.7)	97 (53.3)
I feel less of a man because of reproductive problems	163 (84.5)	23 (11.9)	5 (2.6)	1 (0.5)	1 (0.5)
An illness/disease has affected my ability to have children	131 (70.1)	22 (11.8)	21 (11.2)	5 (2.7)	8 (4.3)
I am angry that my ability to have children has been affected	167 (87.9)	19 (10)	3 (1.6)	-	1 (0.5)
I am able to talk openly about my fertility	64 (35)	10 (5.5)	31 (16.9)	25 (13.7)	53 (29)
Others are to blame for reproductive problems	178 (94.7)	5 (2.7)	3 (1.6)	-	2 (1.1)
I am sad that my ability to have children has been affected	153 (80.5)	28 (14.7)	7 (3.7)	2 (1.1)	-
I was in control over my reproductive future	108 (57.7)	14 (7.5)	18 (9.6)	22 (11.8)	25 (13.4)
I feel guilt about my reproductive problems	178 (93.2)	11 (5.8)	2 (1)	-	-
I have mourned the loss of my ability to have children	169 (89.4)	11 (5.8)	5 (2.6)	4 (2.1)	-
I blame myself for my reproductive problems	183 (95.8)	6 (3.1)	2 (1)	-	-
I am frustrated that my ability to have children has been affected	169 (88.9)	17 (8.9)	2 (1.1)	2 (1.1)	-
I am less satisfied with my life because of reproductive problems	174 (90.6)	14 (7.3)	1 (0.5)	1 (0.5)	2 (1)

## Data Availability

The data presented in this study are available on request from the corresponding author. The data are not publicly available due to privacy issues.
